# Nonalcoholic steatohepatitis increases plasma retention of sorafenib-glucuronide in a mouse model by altering hepatocyte hopping

**DOI:** 10.1016/j.apsb.2024.09.004

**Published:** 2024-09-13

**Authors:** Erica Toth, Hui Li, Kayla Frost, Paxton Sample, Joseph Jilek, Siennah Greenfield, Dahea You, Danielle Kozlosky, Michael Goedken, Mary F. Paine, Lauren Aleksunes, Nathan Cherrington

**Affiliations:** a University of Arizona, Tucson, AZ 85721, USA; b Rutgers University, Piscataway, NJ 08854, USA; c Washington State University, Spokane, WA 99202, USA

**Keywords:** Nonalcoholic steatohepatitis, Hepatocyte hopping, Sorafenib, Sorafenib glucuronide, Drug disposition, ADME, Transport, Knockout mouse

## Abstract

Hepatocyte hopping is the hepatocyte-to-sinusoid-to-hepatocyte shuttling that increases the efficiency of hepatic elimination of xenobiotics. This phenomenon is mediated *via* efflux of hepatic metabolites by Mrp3 and reuptake by Oatp transporters in sequential hepatocytes until eventual biliary efflux by Mrp2. Sorafenib-glucuronide (SFB-G), the major metabolite of sorafenib (SFB), undergoes hepatocyte hopping, leading to efficient biliary elimination. Nonalcoholic steatohepatitis (NASH) alters the functioning of transporters involved in hepatocyte hopping. The purpose of this study was to quantify the effect of NASH on the three drug disposition processes of hepatocyte hopping. Male FVB and C57BL/6 wild-type (WT), *Oatp1a/1b* cluster knockout (O^−/−^), and Mrp2 knockout (*Mrp2*^−/−^) mice were fed a methionine and choline deficient (MCD) diet to induce NASH. Mice were administered 10 mg/kg SFB *via* oral gavage and concentrations of SFB and SFB-G in plasma quantified using liquid-chromatography tandem mass spectrometry. Compared to WT, plasma area under the concentration-time curve (AUC) of SFB-G increased by 108-fold in the O^–/–^-C group and by 345-fold in the *Mrp2*^–/–^-C group. In the WT-NASH group, up-regulation of Mrp3 and decreased Mrp2 function, along with reduced Oatp uptake, elevated SFB-G AUC by 165-fold. SFB-G AUC in the O^–/–^-NASH group increased by 108-fold compared to WT-C (3.2-fold compared to O^–/–^-C). SFB-G AUC in the *Mrp2*^–/–^-NASH group increased by 450-fold (1.2-fold compared to Mrp2^–/–^-C). Taken together, the mislocalization of Mrp2 in NASH is a major contributor to the decrease in SFB-G biliary efflux, but decreased Oatp uptake and enhanced sinusoidal efflux also limit the contribution of downstream hepatocytes, resulting in plasma retention that recapitulates the altered pharmacokinetics observed in human NASH.

## Graphical abstract

Nonalcoholic steatohepatitis (NASH) markedly impedes hepatocyte hopping by altering the expression of key hepatic xenobiotic transporters, leading to plasma accumulation of xenobiotics and increasing the risk of toxicity.

**Figure:**
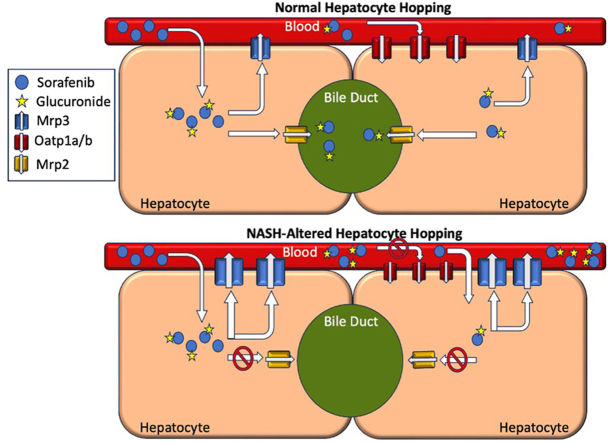

## 1 Introduction

Nonalcoholic steatohepatitis (NASH) is the hepatic manifestation of metabolic syndrome, and disease progression from nonalcoholic fatty liver disease (NAFLD) to NASH is characterized by fibrosis, inflammation, and hepatocellular injury^1^. The worldwide prevalence of NASH is approximately 1.5%–6.5%^2^. Progression to NASH is also complicated by substantive alterations to gene expression, including genes that are responsible for absorption, disposition, metabolism, and excretion processes, such as xenobiotic transporters and drug metabolizing enzymes^3^. These alterations can drastically change the disposition and toxicity of xenobiotics, potentially resulting in adverse drug reactions (ADRs). As nearly 1 in 20 hospitalized patients experience ADRs in the United States^4^, identifying factors that contribute to variations in drug response is critical to improve public health.

Sorafenib (SFB) is a kinase inhibitor used to treat renal cell carcinoma, hepatocellular carcinoma, and radioactive iodine-resistant thyroid cancer. Patient response to SFB treatment is highly variable^5^^,^^6^, although metabolic pathways such as cytochrome P450 (CYP) 3A4-mediated formation of SFB-*N*-oxide and UDP-glucuronosyltransferase (UGT) 1A9-mediated formation of SFB-glucuronide (SFB-G) have been reasonably well characterized and cannot explain the origin of the discrepancy in response^7^^,^^8^. This variability presents difficulties in predicting patients at risk for ADRs. Previously, *Oatp1a/1b* (organic anion transporting polypeptide) cluster knockout mice, which lack several basolateral hepatic uptake transporters, were reported to exhibit increased SFB-G plasma concentrations after oral administration (10 mg/kg) of SFB^9^. Glucuronides such as SFB-G and bilirubin-glucuronide undergo a shuttling process into and out of hepatocytes, mediated through basolateral uptake *via* OATP/Oatp transporters and basolateral excretion *via* MRP3/Mrp3 (multidrug resistance-associated protein) and other unidentified efflux transporters. This process, described by Vasilyeva et al.^9^ as ‘hepatocyte hopping’, is a physiological process that allows for relatively efficient hepatic elimination through multiple opportunities at the excretion site. During this process, glucuronides are 1) returned to sinusoidal blood through MRP3 or 2) secreted into bile through MRP2. Each subsequent hepatocyte that the glucuronide encounters through 3) downstream OATP uptake will afford another opportunity for excretion by MRP2. Thus, if biliary secretion in upstream hepatocytes is saturated, due to either incidental inhibition or overload, biliary secretion is distributed more evenly across the entire lobule, and glucuronidated substrates can still be eliminated^10^.

NASH is known to alter the disposition of drugs through alteration of hepatic xenobiotic transporter expression and function^11^^,^^12^. Among the affected transporters are OATP, MRP2, and MRP3, all of which play critical roles in the hepatocyte hopping of glucuronides. Given previous data on the effects of NASH on xenobiotic disposition, hepatocyte hopping of SFB-G was hypothesized to be disrupted by NASH, resulting in increased plasma concentrations of SFB-G. This study aimed to determine the impact of NASH on the three molecular mechanisms of SFB-G hepatocyte hopping using a methionine-choline deficient (MCD) diet mouse model of NASH in combination with mouse knockout models to isolate the effect of disease on hepatocellular shuttling.

## 2 Materials and methods

### 2.1 Reagents

SFB was purchased from Sigma–Aldrich (St. Louis, MO, USA). SFB-methyl-*d*_3_ was purchased from Santa Cruz Biotechnology (Dallas, TX, USA). SFB-G standard was generously donated by Dr. Sharyn Baker from The Ohio State University (Columbus, OH, USA). Cremophor EL, UPLC-grade acetonitrile, and UPLC-grade water were purchased from Sigma–Aldrich. ReadyScript cDNA synthesis kit, KiCqStart™ SYBR green qPCR master mix, and PCR primers for *Oatp1a1*, *Oatp1a4*, *Oatp2b1*, *Oatp1b2*, *Mrp2*, *Mrp3*, and *β-*actin were obtained from Sigma–Aldrich. RNA Bee isolation reagent was obtained from Amsbio (Cambridge, MA, USA). Synthetic peptides for targeted proteomic analysis were purchased from AnaSpec, Inc. (Fremont, CA, USA).

### 2.2 Animals

Male FVB wild-type and *Oatp1a/1b*^−/−^ mice from Jackson Laboratory (Bar Harbor, ME) at 8 weeks of age were housed in a standard 12-h light/dark cycle in the University of Arizona animal care facility. *Abcc2*^−/−^ mice were obtained from Taconic and backcrossed by the Aleksunes lab at Rutgers University to a C57BL/6 background as previously described^13^. Male C57BL/6 mice were purchased from Charles River Laboratories (Wilmington, MA, USA). The mice were fed either a methionine and choline sufficient (control) diet or a MCD diet from Dyets, Inc. (Bethlehem, PA, USA) for 6 weeks to induce NASH. After 6 weeks of diet, a stock solution of 2.5 mg/mL of SFB was made by dissolving SFB in a 1:1 mixture of Cremophor EL and 100% ethanol, heating to 40 °C in a water bath to aid in dissolution, then diluting 1:4 in deionized water. The animals were administered an oral gavage of 10 mg/kg of SFB (5 mL/kg). Blood collections were taken *via* submental bleed at 20 min and 1, 2, 4, and 8 h after SFB administration and collected into heparinized tubes. Plasma was obtained *via* centrifugation at 3000×*g* for 5 min. After the final time point, animals were euthanized and terminal liver was collected. Plasma samples were stored at −80 °C, and tissue samples were flash-frozen in liquid nitrogen before storing at −80 °C. The animal study was approved by the University of Arizona Animal Care and Use Committee and the Rutgers University Animal Care and Use Committee.

### 2.3 Sorafenib and sorafenib-glucuronide quantification

The method for quantification of SFB and SFB-G was adapted from previously established methods^9^^,^^14^. The Arizona Laboratory for Emerging Contaminants provided a Waters (Milford, MA, USA) Micromass Quattro Premier XE tandem mass spectrometer coupled with an Acquity UPLC. The mobile phase consisted of UPLC-grade water with 0.1% formic acid (A) and acetonitrile with 0.1% formic acid (B). A Waters Acquity UPLC BEH C18 column (1.7 μm, 2.1 mm × 50 mm) was used for separation and set to a flow rate of 0.3 mL/min. The UPLC gradient began at 60% A, then decreased to 35% A from 1.0 to 1.70 min and held until 2.50 min. After 2.50 min, A decreased to 5% and held until 4.50 min before returning to 60% A and equilibrating for 1 min. The injection volume was 10 μL. SFB was detected at *m/z* 465.1>270, SFB-G was detected at *m/z* 641.2>465.1, and SFB-methyl-*d*_3_ (internal standard) was detected at *m/z* 468>255. The assay was validated between 10 and 10,000 ng/mL for SFB and 10–30,000 ng/mL for SFB-G, with the lowest limit of these ranges being the lower limit of quantification (LLOQ). Raw data were processed using the Waters MassLynx software.

### 2.4 Tissue preparation

Liver tissue was prepared for LC–MS/MS quantification of SFB-G. Tissue (200 mg) was homogenized in 400 μL of cold 4% (*w*/*v*) bovine serum albumin in UPLC-grade water. Twenty μL of homogenate was placed into a 1.5 mL microcentrifuge tube and combined with 5 μL of internal standard. Forty μL of acetonitrile was added to precipitate proteins, and the mixture was vortexed vigorously. The homogenate was centrifuged at 10,000×*g*, and the supernatant was pipetted into a 250 μL glass insert and diluted with mobile phase to a total volume of 120 μL.

### 2.5 Protein preparation

Crude membrane preparations were prepared from liver tissue samples. Approximately 100 mg of tissue was homogenized in 1 mL of cold sucrose Tris buffer (10 mmol/L Tris base and 250 mmol/L sucrose) with commercial protease inhibitor (Roche, Indianapolis, IN, 1 tablet per 50 mL buffer). Homogenates were centrifuged at 10,000×*g* for 20 min to remove nuclei, and the supernatant was decanted into a second set of ultracentrifuge tubes. The supernatant was spun at 100,000×*g* for 60 min to pellet the membranes. Membrane pellets were rinsed with buffer, then resuspended in 100 μL buffer and stored at −80 °C. Protein concentrations were determined *via* the BCA assay (Thermo Fisher, Waltham, MA, USA).

### 2.6 LC–MS/MS quantification of transporter proteins by surrogate peptides

To evaluate protein expression by surrogate peptide quantification, 300 μg of membrane protein per sample was diluted into 100 mmol/L ammonium bicarbonate and 3.7% sodium deoxycholate. Proteins were denatured with 6 mmol/L dithiothreitol at 95 °C for 5 min, gradually returned to room temperature, then alkylated with 15 mmol/L of iodoacetamide in the dark. The alkylation reaction was quenched with 20 mmol/L dithiothreitol and proteins were pelleted by sequential addition of 0.5 mL ice-cold methanol, 0.1 mL chloroform, and 0.4 mL water and centrifuged at 16,000×*g* for 5 min. The protein pellet was resuspended in 100 μL of 3.7% sodium deoxycholate and 50 mmol/L ammonium bicarbonate and then digested with 3 μg sequencing-grade trypsin (Promega, Madison WI, USA) in a water bath at 37 °C overnight. The following day, the reaction was quenched with 0.4% formic acid in water and samples were centrifuged at 16,000×*g* for 30 min. The supernatant (peptide fraction) was desalted and concentrated with Waters Oasis MCX strong cation exchange cartridges (Milford, MA, USA) per the manufacturer instructions. The eluted peptides were dried down using a centrivap, reconstituted in LC mobile phase, and 10 μL was injected onto a Waters Acquite UPLC BEH C18 column (2.1 mm × 50 mm, 1.7 μm bead diameter). Separation was achieved using an Agilent 1290 Infinity II UPLC (Agilent Technologies, Santa Clara, CA, USA) with a binary gradient flow of water with 0.1% formic acid (mobile phase A) and 90:10 acetonitrile:water with 0.1% formic acid (mobile phase B) at 0.2 mL/min flow rate as follows: 5.5% B (0–2 min), 5.5%–33% B (2–22 min), 33% B (22–24 min), 100% B (24–26 min), 5.5% B (26–28 min). Eluted peptide analytes were detected on a Sciex QTrap 6500+ triple quadrupole mass spectrometer (Framingham, MA, USA) and operated in positive electrospray ionization mode with the following source parameters: source voltage, 5.5 kV; curtain gas, 20 psi; nebulizer gas, 50 psi; turbo gas, 25 psi. Doubly charged surrogate peptide parent ions [M+2H]^2+^ and singly charged fragment ions [M+H]^+^ were detected by multiple reaction monitoring (MRM) using a declustering potential of 50 V, entrance potential of 10 V, collision energy of 25 V, and collision cell exit potential of 15 V for all analytes. Surrogate peptide sequences and mass transitions were selected using Skyline software^15^ and unique tryptic peptides were filtered against homologous sequences within the host genome by MS-Homology within Protein Prospector (http://prospector.ucsf.edu/prospector/). Raw data were analyzed and quantified using Analyst 1.6.2 (SCIEX, Ontario, Canada) and normalized to Na/K ATPase intensity.

### 2.7 Quantitative reverse transcription-PCR

RNA was isolated from liver tissue using RNA Bee isolation reagent (Amsbio, Cambridge, MA, USA). Tissue (200–300 mg) was added to 4 mL of RNA Bee and homogenized *via* a probe homogenizer. Following the manufacturer's protocol, the RNA pellet was reconstituted in 250 μL of diethylpyrocarbonate (DEPC) water per 100 mg of tissue, then stored at −80 °C. RNA concentrations were quantified using a NanoDrop 200 UV–Vis spectrophotometer (Thermo Fisher Scientific, Waltham, MA, USA), and final RNA preparations had a 260/280 quality ratio between 1.6 and 1.9. ReadyScript® cDNA synthesis kit (Sigma–Aldrich, St Louis, MO, USA) was used to prepare cDNA. Each reaction well contained 2 μL of cDNA template, 100 nmol/L of forward and reverse primers, 1 × KiCqStart™ SYBR® green master mix, and nuclease free water up to 20 μL, run in duplicate. mRNA concentrations were measured using an ABI StepOnePlus Real Time PCR system with a standard SYBR® green PCR cycling profile. The delta-delta C_T_ method was used to determine fold changes.

### 2.8 Histopathology

Paraffin-embedded liver sections were stained with hematoxylin and eosin (H&E) and examined by a board-certified veterinary pathologist. Tissues were incidence and severity scored using an established rodent NASH system^16^ with endpoints including steatosis, necrosis, inflammation, hyperplasia, and biliary hyperplasia. Representative digital images were acquired using a light microscope at 40× magnification.

### 2.9 Statistics

All results are represented as the mean ± standard deviation (SD). Two-way ANOVA analyses with Bonferroni post-test were used to compare between groups. Student's *t*-test was used to compare between two groups. Each group contained *n* = 6 animals.

## 3 Results

### 3.1 Effects of diet-induced NASH and genetic knockouts on histopathology

H&E stained liver sections were examined under a light microscope at 40× magnification to determine disease severity. NASH hallmarks were observed in mice fed an MCD diet for 6 weeks, including steatosis, inflammation, fibrosis, necrosis, and biliary hyperplasia (Supporting Information Fig. S1). These observations recapitulated the disease reported in human patients and is consistent with previous findings^16^^,^^17^.

### 3.2 Effects of diet-induced NASH and Oatp1a/1b cluster knockout on plasma concentration of sorafenib-glucuronide

**Figure 1:**
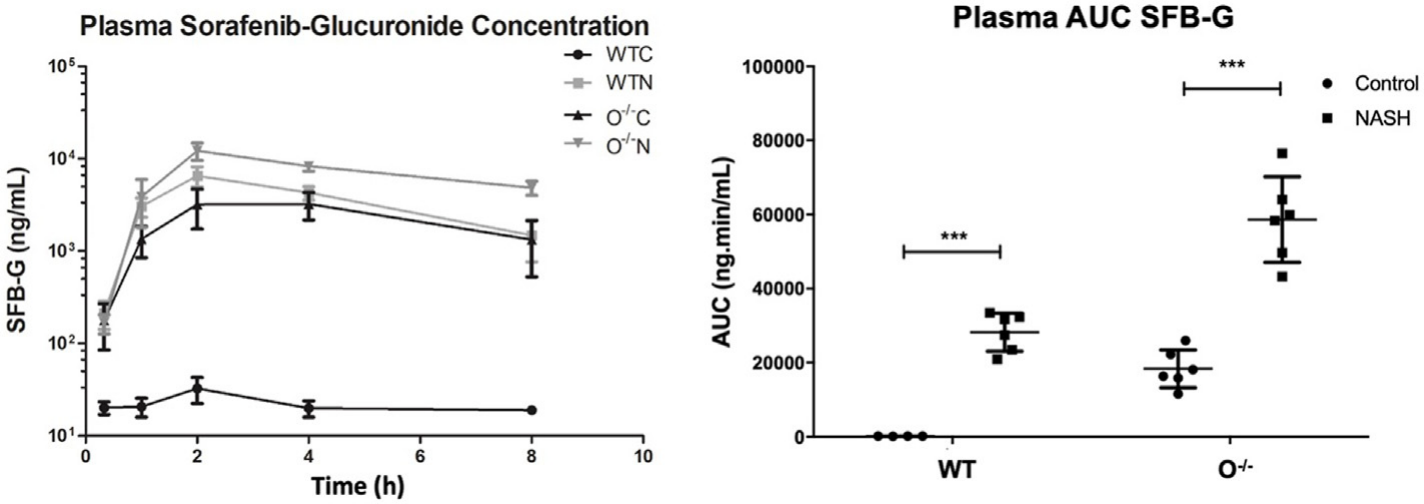
Effects of Oatp1a/1b cluster knockout and NASH on plasma sorafenib-glucuronide concentration. Plasma concentrations were measured over 8 h after administration of a 10 mg/kg oral dose of sorafenib. Plasma AUC represents mean ± SD, *n* = 6. ∗*P* ≤ 0.05, ∗∗*P* ≤ 0.01, ∗∗∗*P* ≤ 0.001.

The effects of NASH and *Slco1a/1b* cluster knockout on SFB and SFB-G systemic exposure in mice after oral administration of SFB were determined over the course of 8 h (Fig. 1). Compared to wild type control (WT-C) mice, which had no SFB-G in plasma due to proper transport function, the plasma area under the concentration *versus* time curve (AUC) of SFB-G increased in WT-NASH mice by 165-fold and in O^–/–^-C mice by 108-fold. Compared to O^–/–^-C mice, SFB-G AUC increased in O^–/–^-NASH mice by 3.2-fold. No statistically significant change was observed between the SFB AUCs of any group (Supporting Information Fig. S2). These data indicate that Oatp function is one determinant in the disposition of SFB-G and that the NASH-induced alterations to transporter function impact SFB-G plasma concentrations.

### 3.3 Effects of diet-induced NASH Mrp2 knockout on sorafenib-glucuronide disposition

**Figure 2:**
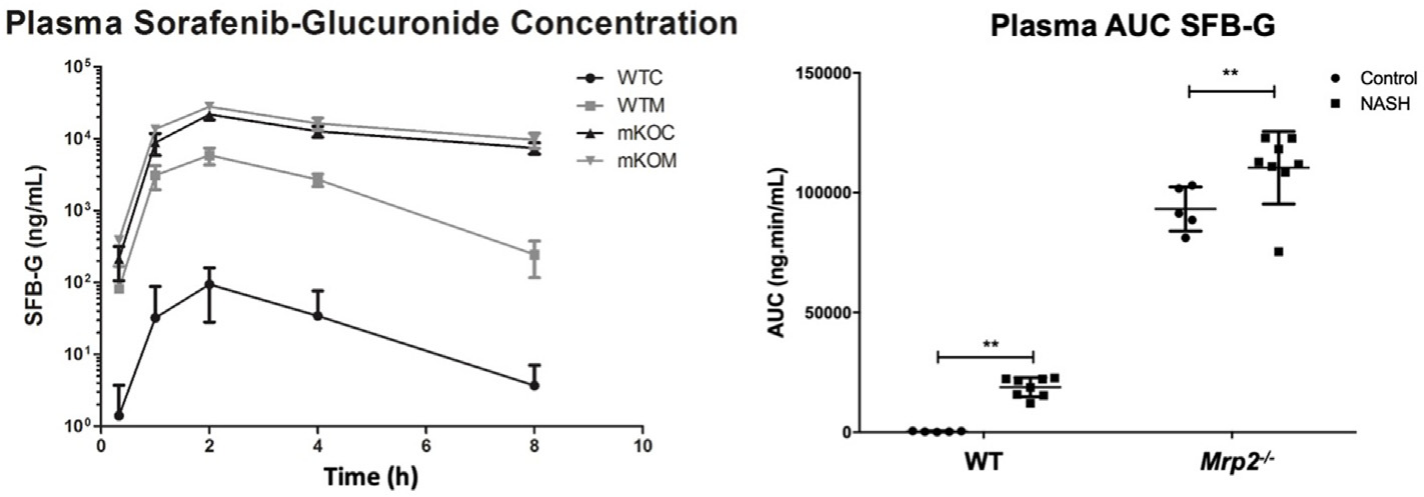
Effects of Mrp2 knockout and NASH on plasma sorafenib-glucuronide concentration. Plasma concentrations were measured over 8 h after administration of 10 mg/kg oral dose of sorafenib. Plasma AUC represents mean ± SD, *n* = 6. ∗*P* ≤ 0.005, ∗∗*P* ≤ 0.01, ∗∗∗*P* ≤ 0.001.

The effects of NASH and *Mrp2* knockout on SFB-G systemic exposure in mice after oral administration of SFB were determined over a period of 8 h (Fig. 2). Compared to WT-C mice, SFB-G AUC in WT-NASH mice increased by 68-fold and in *Mrp2*^–/–^-C mice by 335-fold. Compared to *Mrp2*^–/–^-C mice, SFB-G AUC increased in *Mrp2*^–/–^-NASH mice by 1.2-fold. No statistically significant change was observed between the SFB AUCs of any group (Fig. S2). These data indicate that Mrp2 function is a major, but not sole, determinant of SFB-G disposition.

### 3.4 Effects of diet-induced NASH and Oatp1a/1b cluster knockout on liver concentrations of sorafenib-glucuronide

**Figure 3:**
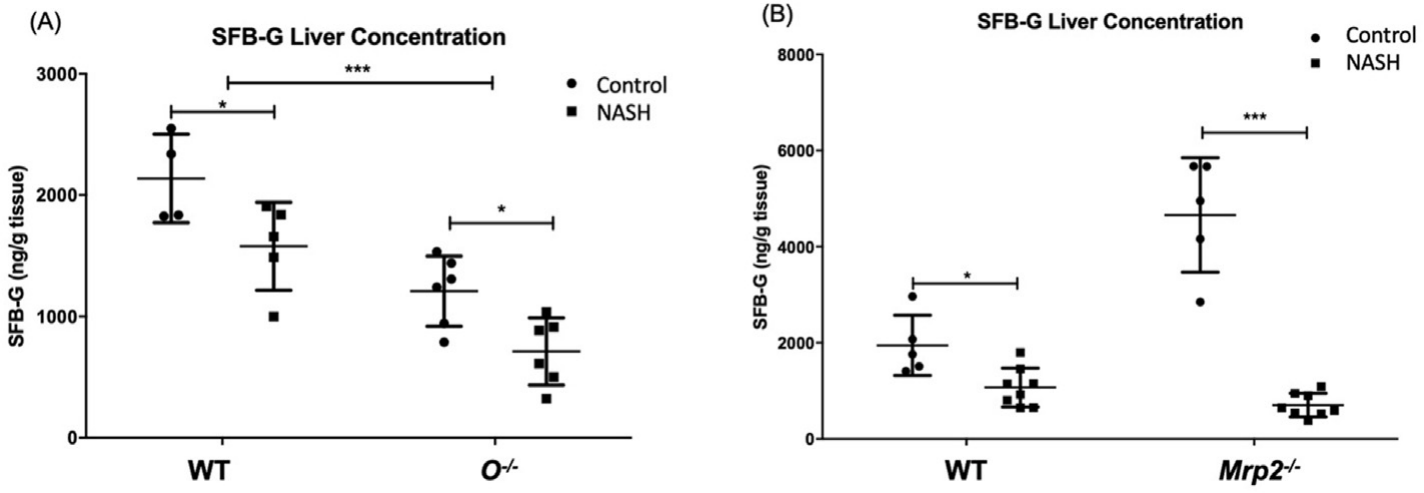
Effects of Oatp1a/1b cluster knockout and Mrp2 knockout and NASH on liver tissue sorafenib-glucuronide concentration. SFB-G concentration in liver tissue at the end of the experimental period (8 h) in (A) *Slco1a/1b* cluster knockout mice and (B) *Abcc2* knockout mice after oral administration of a 10 mg/kg oral dose of sorafenib. Data represent mean ± SD, *n* = 6. ∗*P* ≤ 0.05, ∗∗*P* ≤ 0.01, ∗∗∗*P* ≤ 0.001.

Hepatic concentrations of SFB-G were determined at the end of the experimental period (8 h) to assess the effect of diet-induced NASH and *Slco1a/1b* cluster knockout on SFB-G disposition in mice after oral administration of SFB (Fig. 3A). Compared to WT-C mice, the liver concentration of SFB-G decreased in WT-NASH mice by 24%; compared to O^–/–^-C mice, SFB-G concentration decreased in O^–/–^-NASH mice by 43%. Comparing the O^–/–^-C with the WT-C mice to assess the impact of loss of *Oatp1a/1b* function alone, the liver SFB-G concentration in the O^–/–^-C group decreased by 43%. Comparing the O^–/–^-NASH to the WT-C mice, liver SFB-G concentration decreased by 62% (Fig. 3A). No significant changes were found in the concentrations of SFB between any of the groups (Supporting Information Fig. S3). These data indicate that Oatp function contributes significantly to the uptake of SFB-G into hepatocytes, and loss of function results in lower hepatic concentrations. The alterations to transporter function that occurs during NASH also impede Oatp-mediated uptake and retention in hepatocytes, resulting in reduced liver concentrations.

### 3.5 Effects of diet-induced NASH and Mrp2 knockout on liver concentrations of sorafenib-glucuronide

The effects of diet-induced NASH and *Mrp2* knockout on SFB-G disposition in the liver (Fig. 3B) were determined in the same manner as described for the *Oatp1a/1b* cluster knockout mice. Compared to WT-C mice, the concentration of SFB-G in the liver in WT-NASH mice decreased by 45%; compared to *Mrp2*^−/−^-C mice, SFB-G concentration in *Mrp2*^–/–^-NASH mice decreased by 85%. Comparing the *Mrp2*^−/−^-C with WT-C mice to assess the impact of loss of Mrp2 function alone, the liver SFB-G concentration increased by 2.4-fold. Compared to WT-C mice, liver SFB-G concentration in *Mrp2*^–/–^-NASH mice decreased by 64% (Fig. 3B). No changes were observed in the concentrations of SFB between any of the groups (Fig. S3). These data indicate that Mrp2 function contributes significantly to the efflux of SFB-G from hepatocytes, and loss of function will result in hepatic accumulation of the glucuronide. Loss of function of Mrp2 combined with disruption of hepatocyte hopping due to diet-induced NASH, resulted in an even greater reduction in hepatic accumulation of SFB-G.

### 3.6 Effects of diet-induced NASH and Oatp1a/1b cluster knockout and Mrp2 knockout on xenobiotic transporter gene expression

**Figure 4:**
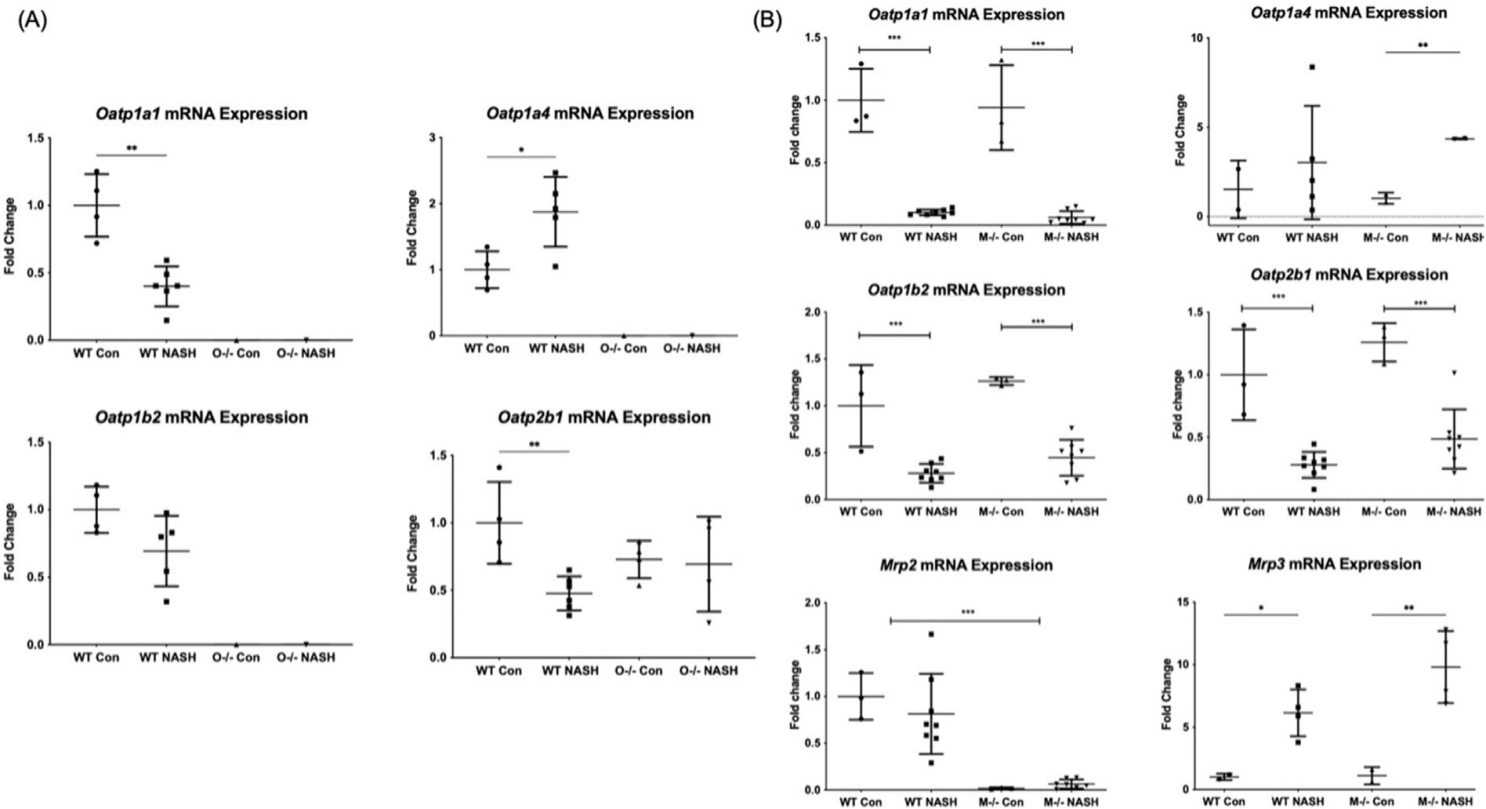
Altered mRNA expression of Oatp and Mrp transporters due to Oatp1a/1b cluster knockout and Mrp2 knockout and diet-induced NASH. mRNA expression of *Oatp* transporters and *Mrp* transporters in (A) *Oatp1a/1b* cluster knockout mice and (B) *Mrp2* knockout mice was determined through qRT-PCR, and fold change was determined using the ΔΔC_T_ method. Data represent mean ± SD, *n* = 6. ∗*P* ≤ 0.05, ∗∗*P* ≤ 0.01, ∗∗∗*P* ≤ 0.001.

mRNA expression of Oatp uptake transporters and Mrp efflux transporters was assessed to determine the impact of NASH, *Slco1a/1b* cluster knockout, and Mrp2 knockout on gene expression (Fig. 4). *Slco1a1* mRNA in WT-NASH mice decreased by 64% compared to control; mRNA was not detected in the O^–/–^-C or O^–/–^-NASH mice (Fig. 4A, upper left panel). *Slco1a1* mRNA in *Mrp2*^–/–^-NASH mice decreased by 90% compared to control (Fig. 4B, upper left panel). *Slco1a4* mRNA increased to 168% of control (Fig. 4B, upper right panel), to 181% in Mrp2^–/–^-NASH mice compared to control (Fig. 4B, upper right panel), and was not detected in the *Slco1a/1b* knockout groups (Fig. 4A, upper right panel). *Slco1b2* mRNA decreased to 63% of control in the WT-NASH group, to 44% of control in the *Mrp2*^–/–^-NASH group, and was not detected in either of the O^−/−^ groups (Fig. 4A and B, middle left panels). *Slco2b1* mRNA decreased to 52%, 58%, and 56% of control in the WT-NASH, O^–/–^-C, and O^–/–^-NASH groups, respectively. *Slco2b1* mRNA reduced to 40% of control in the *Mrp2*^–/–^-NASH group (Fig. 4A and B, middle right panels). There were no statistically significant alterations to the mRNA expression of *Abcc2* between the WT and NASH groups (Fig. 4B, lower left panel). *Abcc3* mRNA was significantly altered in the *Mrp2*^–/–^-NASH group, where it increased to 479% of control (Fig. 4B, lower right panel).

### 3.7 Effects of diet-induced NASH and Oatp1a/1b cluster knockout and Mrp2 knockout on xenobiotic transporter protein expression

**Figure 5:**
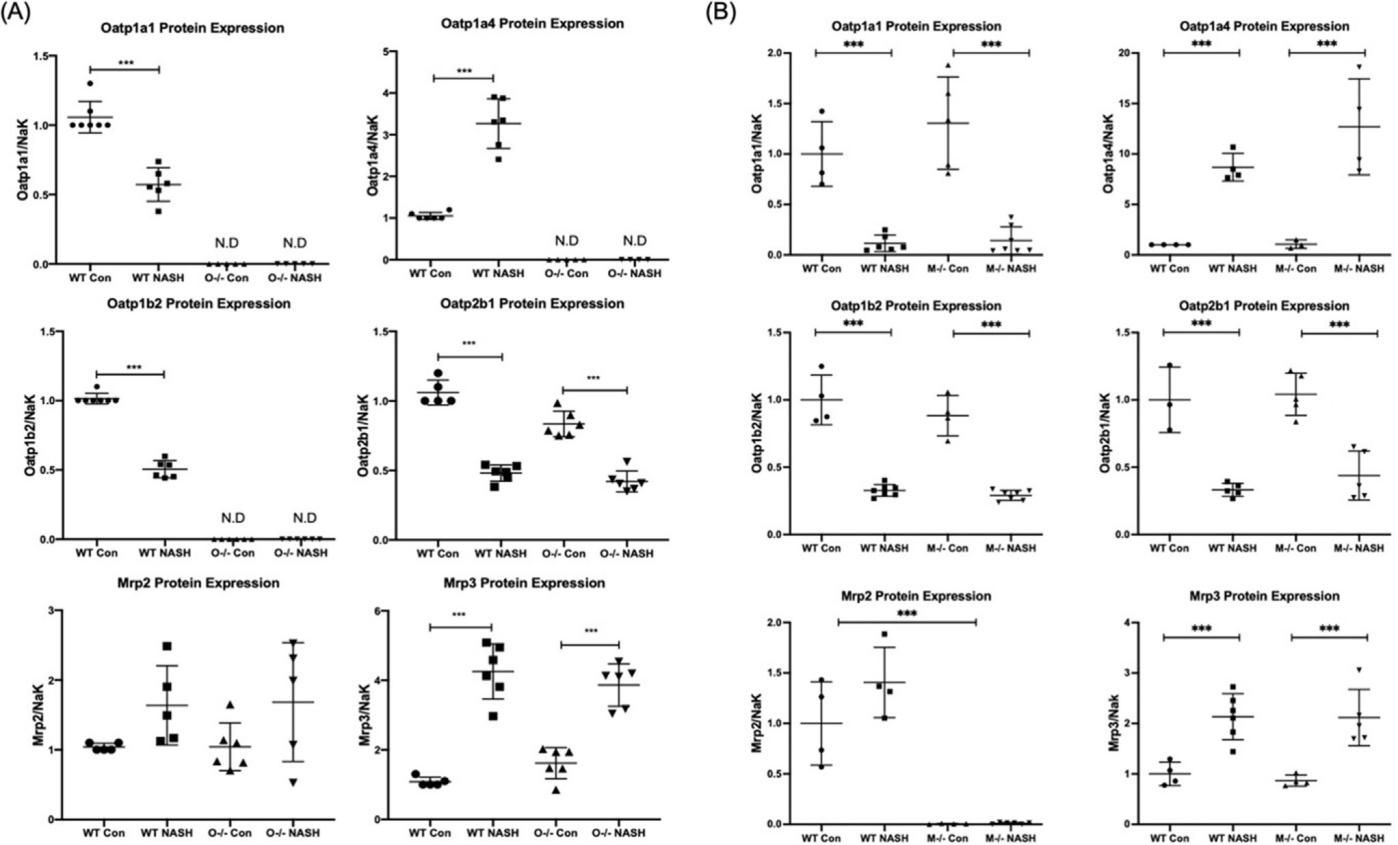
Altered protein expression of Oatp and Mrp transporters due to Oatp1a/1b cluster knockout and Mrp2 knockout and diet-induced NASH. Relative protein expression in (A) *Slco1a/1b* cluster knockout mice and (B) *Abcc2* knockout mice was determined by proteomic analysis using LC–MS/MS and normalized to Na^+^/K^+^-ATPase expression with Mrp2 heavy labeled internal standard. Data represent mean ± SD, *n* = 6. ∗*P* ≤ 0.05, ∗∗*P* ≤ 0.01, ∗∗∗*P* ≤ 0.001.

Relative protein concentrations of Oatp1a1, Oatp1a4, Oatp1b2, Oatp2b1, Mrp2, and Mrp3 were determined *via* LC–MS/MS and normalized to Na^+^/K^+^-ATPase (Fig. 5). Oatp1a1 protein expression decreased to 57% of control in the WT-NASH group, to 11% of control in the *Mrp2*^–/–^-NASH group, and was not detected in the *Slco1a/1b* cluster knockout animals (Fig. 5A and B, upper left panels). Oatp1a4 protein expression increased to 327% of control in the WT-NASH group, to 806% of control in the *Mrp2*^–/–^-NASH group, and was not detected in *Oatp1a/1b* cluster knockout animals (Fig. 5A and B, upper right panels). Oatp1b2 protein expression decreased to 51% of control in the WT-MCD group, to 33% of control in the *Mrp2*^–/–^-NASH group, and was not detected in the *Slco1a/1b* cluster knockout animals (Fig. 5A and B, middle left panels). Oatp2b1 protein expression decreased to 48% of control in the WT-MCD group, to 42% of control in the *Mrp2*^–/–^-NASH group, and to 42% of control in the O^–/–^-NASH group (Fig. 5A and B, middle right panels). Mrp3 protein expression increased to 425% of control in the WT-NASH group, to 245% of control in the *Mrp2*^–/–^-NASH group, and to 386% of control in the O^–/–^-NASH group (Fig. 5A and B, lower right panels). The protein expression of Mrp2 did not significantly change between any group, and no Mrp2 protein was detected in the *Abcc2* knockout groups (Fig. 5A and B, lower left panels).

## 4 Discussion

Following UGT-mediated conjugation of SFB after oral administration, the disposition of SFB-G is mediated by sinusoidal efflux through Mrp3, biliary efflux *via* Mrp2, and subsequent sinusoidal uptake through Oatp transporters. Progression of NAFLD to NASH includes alterations to hepatic enzymes and transporters responsible for drug disposition in humans^3^. These alterations result in nearly identical changes to the functional disposition of several drugs in both human patients and in rodent models of NASH. The three mechanisms of hepatocyte hopping are concordantly regulated in both human NASH and MCD diet-induced NASH, and drugs that rely on any one of these three mechanisms show strikingly similar changes in disposition. In the case of acetaminophen (APAP), after the intravenous administration of a nontoxic dose (1 mmol/kg), biliary concentrations of the three major metabolites (APAP-sulfate, APAP-glucuronide, APAP-glutathione) were significantly decreased in rats with diet-induced NASH. Additionally, the Mrp3 substrate APAP-glucuronide was elevated in the plasma of rats with NASH when compared to the other metabolites and was 80% higher in urine^18^. The increased APAP-glucuronide concentrations in serum and urine observed in the MCD rodent model also presented only in patients with NASH. This shift indicated that the increased expression of Mrp3 and mislocalization of Mrp2 in the MCD model of NASH is identical to that observed in human NASH patients^19^. There was a similar agreement between the changes to morphine pharmacokinetics in the MCD rat model and human patients, showing an increase in systemic serum morphine-glucuronide exposure and an increase in serum exposure of morphine-3-glucuronide due to decreased biliary efflux and enhanced sinusoidal efflux of the glucuronide^20^, ^21^, ^22^. While the extrapolation of animal data to predict toxicity in humans remains challenging, the MCD diet-induced model of NASH offers utility in that it closely resembles human NASH histology and recapitulates the molecular mechanisms of hepatic transporter alterations observed in human patients^17^^,^^23^.

Efflux transporters are known to markedly impact drug disposition. In the MCD rodent model of NASH, the disease causes an increase in the protein expression of Mrp3 and no change in the overall protein expression of Mrp2^17^^,^^23^; however, the function of Mrp2 is significantly impaired due to mislocalization of the transporter from the bile canalicular membrane^11^. These alterations have been shown to impact the distribution of various xenobiotics such as pemetrexed, methotrexate, and ezetimibe^24^, ^25^, ^26^. As SFB-G is known to be a substrate for Oatp uptake transporters and Mrp2, the functional status of these transporters will significantly impact the disposition of this metabolite. While Mrp3 is also known to contribute to the sinusoidal efflux of SFB-G, full genetic knockout of this protein has only a modest impact on SFB-G pharmacokinetics^9^, implicating the participation of an unknown transporter in this process.

In addition to the ATP-binding cassette (ABC) efflux transporters, Oatp uptake transporters play a crucial role in the hepatocyte hopping process and are altered during the progression of NASH. The MCD diet has been shown to recapitulate the alterations observed in human disease and alter the protein and mRNA expression of several Oatp proteins, including Oatp1a1, Oatp1a4, Oatp1b2, and Oatp2b1. In the rodent model, the mRNA expression of *Slco1a1*, *Slco1b2*, and *Slco2b1* has been shown to decrease, whereas the expression of *Slco1a4* is up-regulated. Similarly, the protein expression of Oatp1a1, Oatp1b2, and Oatp2b1 decrease, while expression of Oatp1a4 increases^27^. Disease-related transporter impairment is known to significantly reduce the hepatic uptake of ^99m^Tc-mebrofenin, a known substrate of OATP1B1 and OATP1B3, in human patients^28^. It has also been previously established that alterations to Oatp uptake transport can have a significant impact on the disposition of SFB and SFB-G^29^, as well as other xenobiotics including SN-38, the active metabolite of irinotecan^10^^,^^29^. In the MCD rodent model, the interaction of NASH and *Slco1b2* knockout was shown to significantly elevate plasma and muscle concentrations of pravastatin, potentially leading to an increase in toxicity^27^.

There is evidence that the glycosylation status of transporter proteins are altered during NASH, and the presence or absence of these modifications impact the function of the protein. *N*-linked glycosylation of transporter proteins serves several different purposes, including stability, function, and localization, and membrane-bound proteins require proper glycosylation for trafficking^30^^,^^31^. Several genes associated with *N*-linked glycosylation are downregulated in human NASH, indicating a possible perturbation; this observation is supported by an increase in unglycosylated OATP1B1, OATP1B3, OATP2B1, and NTCP in human NASH patients^32^. Impairment of *N*-linked glycosylation may be one mechanism by which NASH disrupts xenobiotic transporter function.

The disposition of SFB-G can also be affected by the activity of UGTs, although it is unlikely that UGT1A9 metabolism is a significant contributor to the variability in SFB-G disposition observed in NASH. UGT1A9 is the major isoform responsible for the glucuronidation of SFB^33^, and some polymorphisms of UGT1A9 have been associated with enhanced risk of toxicity in patients prescribed SFB^34^. In human NASH patients, hepatic UGT1A9 protein expression was downregulated^35^, and a similar decrease was observed in the mRNA expression of this enzyme in steatotic mice^36^. These observations render it unlikely that the alterations to the disposition of SFB-G would be due to the effects of NASH on the expression of the enzyme.

Systemic inflammation and other disease processes could provide the mechanistic basis for the larger category of interindividual variability that cannot be explained by genetics. Disease state, however, can also be a factor that impacts the expression and function of hepatic xenobiotic transporters. Xenobiotic transporters from both the OATP/Oatp and ABCC/Abcc families are known to be significantly altered during the progression of NASH, leading to significant changes in drug disposition. Previous studies have investigated the synergistic effects of gene-by-environment interactions of NASH and hepatic transporter polymorphisms and reported that the interaction between NASH and deleterious polymorphisms can result in significant increases in plasma exposure and potential toxicity of xenobiotics^12^^,^^27^. This study describes the first attempt to isolate the mechanisms involved in hepatocyte hopping and quantitate their impact on SFB-G pharmacokinetics in NASH after oral SFB administration. The effects can be readily observed by the significant elevation in SFB-G plasma concentrations when comparing the WT-NASH group to its control, as well as in the 3.2-fold increase in SFB-G AUC when comparing the O^–/–^-NASH group to the O^–/–^-C group. Disruption of Oatp uptake in the knockout group prevents the reuptake of SFB-G into downstream hepatocytes, prolonging plasma exposure. The additional alterations to Mrp2 and Mrp3 function due to the effects of diet-induced NASH further exacerbate the disruption to hepatocyte hopping, as the loss of Mrp2 function prevents biliary excretion and increased Mrp3 protein expression at the plasma membrane increases the shuttling of SFB-G back into circulation. This can be most readily seen in the Mrp2 knockout groups, where the genetic disruption of Mrp2 alone caused a significant increase in plasma SFB-G concentrations, and the addition of the MCD diet further increased the plasma retention of SFB-G. These data, taken in conjunction with the tissue data that showed an increase in hepatic SFB-G concentrations in *Mrp2* knockout animals but a significant decrease in the knockout MCD group, indicate that the disruption of biliary efflux *via* Mrp2 is not the sole mediator of plasma retention of SFB-G, and that alterations to sinusoidal uptake and efflux during MCD diet-induced NASH contributed to the changes in SFB-G pharmacokinetics. These data confirm that the hepatocellular shuttling of SFB-G is significantly impaired during NASH due to disruption of the three cooperative transport mechanisms of 1) increased Mrp3 sinusoidal efflux, 2) impaired Mrp2 biliary efflux due to mislocalization, as well as 3) decreased Oatp sinusoidal uptake, and quantifies the contribution of these mechanisms towards the overall changes to SFB-G pharmacokinetics. This research elucidates the mechanistic basis behind interindividual variability in SFB-G pharmacokinetics observed in patients undergoing treatment with sorafenib or other drugs that undergo glucuronidation. Disruption of the hepatocyte hopping process during NASH forms the underlying mechanism of pharmacokinetic alterations that have been reported in the progression of this disease and demonstrates the importance of the three major processes of hepatocyte hopping to drug disposition in human patients.

## Appendix A Supporting information

The following is the Supporting Information to this article:
