# Supplementary material for: Nonalcoholic steatohepatitis increases plasma retention of sorafenib-glucuronide in a mouse model by altering hepatocyte hopping
Source: Acta Pharm Sin B. 2024 Sep 13;14(11):4874–82. doi: 10.1016/j.apsb.2024.09.004 (PMC11628858; doi:10.1016/j.apsb.2024.09.004)
Supplement: Multimedia component 1 [file mmc1.pdf]

Supporting Information for

Original article

Nonalcoholic steatohepatitis increases plasma retention of sorafenib-glucuronide in a mouse model by altering hepatocyte hopping

Erica Toth[a], Hui Lia, Kayl[a] Frost[a], Paxton Sample[a], Joseph Jilek[a], Siennah Greenfield[a], Dahea You[b], Danielle Kozlosky[b], Michael Goedken[b], Mary F. Paine[c], Lauren Aleksunes[b], Nathan Cherrington[a]

[a]*University of Arizona, Tucson, AZ 85721, USA*

[b]*Rutgers University, Piscataway, NJ 08854, USA*

[c]*Washington State University, Spokane, WA 99202, USA*

Received 1 March 2024; received in revised form 5 April 2024; accepted 28 May 2024

*Corresponding author.

E-mail address: cherrington@pharmacy.arizona.edu (Nathan Cherrington).

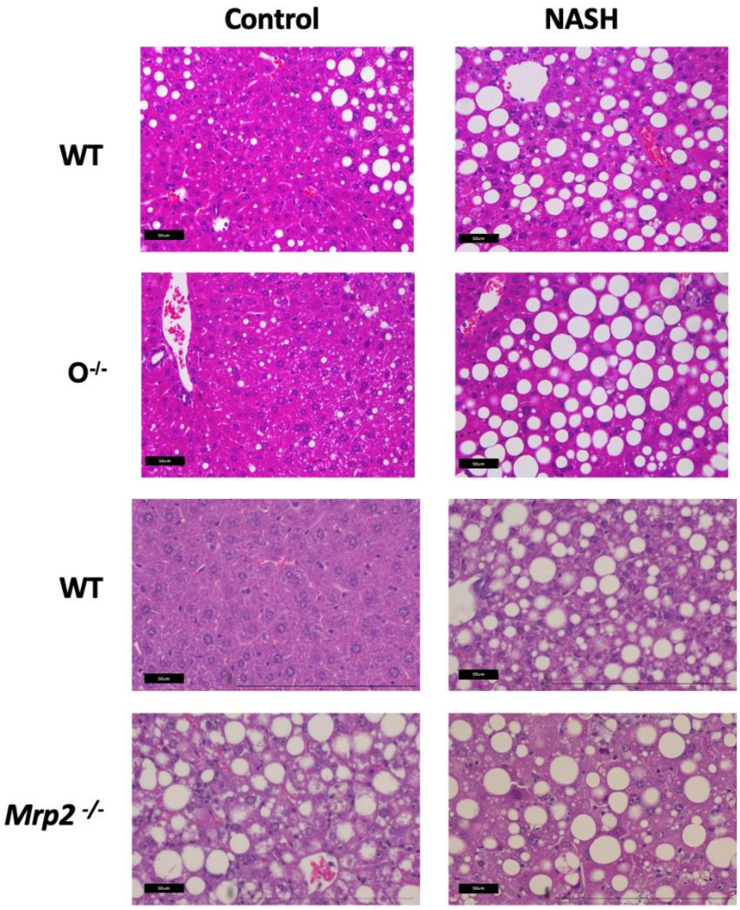

Figure S1. Liver histopathology of control and diet-induced NASH mice. H&E-stained liver sections of control mice and mice fed 8 weeks of MCD diet; WT, Oatp1a/1b cluster KOs, and Mrp2 KOs. Original magnification, 40×. Black bar length, 50 μm.

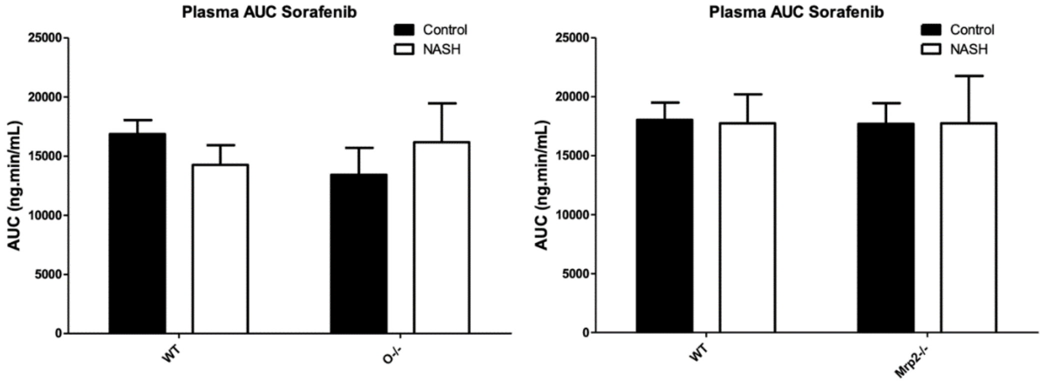

Figure S2. Plasma AUC of SFB in WT, *Oatp1a/b* KO, and *Mrp2* KO. Plasma concentrations of SFB were measured over 8 h after administration of a 10 mg/kg oral dose of sorafenib. Plasma AUC represents mean ± SD, *n*=6.

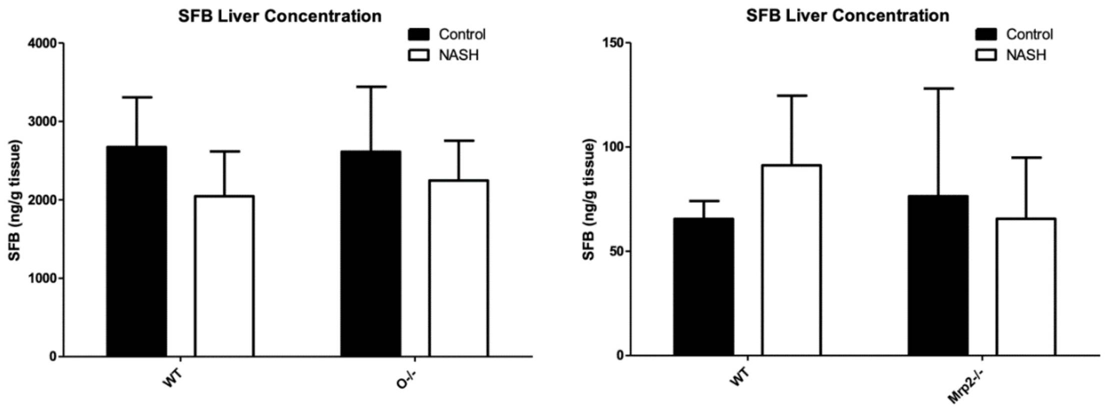

Figure S3. Liver concentration of SFB in WT, *Oatp1a/b* KO, and *Mrp2* KO. SFB concentration in liver tissue at the end of the experimental period (8 h) in *Slco1a/1b* cluster knockout mice and (B) *Abcc2* knockout mice after oral administration of a 10 mg/kg oral dose of sorafenib. Data represent mean mean ± SD, *n*=6.
